# Association between Serum Calcium and First Incident Acute Myocardial Infarction: A Cross-Sectional Study

**DOI:** 10.18502/ijph.v49i7.3581

**Published:** 2020-07

**Authors:** Chi ZHANG, Bangming CAO, Xingmei HUANG, Jian GU, Ming XIA, Xiangjun YANG, Hongxia LI

**Affiliations:** 1. Department of Cardiology, The First Affiliated Hospital of Soochow University, Suzhou 215006, China; 2. Department of Cardiology, Zhejiang Hospital, Hangzhou 310013, China

**Keywords:** Cross-sectional, Coronary artery disease, Acute myocardial infarction, Serum calcium

## Abstract

**Background:**

The role of serum calcium in coronary artery disease (CAD) patients with or without first incident acute myocardial infarction has not been studied previously. This study aimed to assess the relationship between serum calcium and first incident acute myocardial infarction.

**Methods:**

This cross-sectional study was conducted from Jan 2014 to Dec 2016. All the participants were from our database, described in detail elsewhere including 1609 cases and 3252 controls. Multiple logistic regression was carried out to explore the effect of serum calcium on first incident acute myocardial infarction. Interaction between serum calcium and risk factors were evaluated.

**Results:**

Patients with first incident acute myocardial infarction have significantly lower serum calcium concentrations than those without acute myocardial infarction (2.18 (0.21) vs 2.24 (0.19) mmol/L, *P*<0.0001). After adjusting for sex and age, logistic regression showed that serum calcium was significantly associated with first incident acute myocardial infarction (odds ratio (OR): 1.50, 95% confidence interval (CI): 1.41–1.60). Further adjusted for potential confounders, serum calcium was associated with first incident acute myocardial infarction (OR: 1.32, 95% CI: 1.22–1.42). Moreover, the association still existed when patients were divided into subgroups according to gender and age. A significant interaction was found between serum calcium and diabetes mellitus (DM), lipoprotein (a) (Lp (a)), and serum albumin.

**Conclusion:**

Serum calcium was associated with first incident acute myocardial infarction among CAD patients in both sexes and in age categories. This study provides further evidence showing the value of serum calcium levels in clinical practice.

## Introduction

Cardiovascular mortality has fallen dramatically in developed countries during the past four decades because of risk factors management and treatment improvement (1). Despite good progress in the management of patients with cardiovascular disease, mortality has increased in last two decades in China (2). Cardiovascular disease continues to be a leading cause of death (3). First incident acute myocardial infarction (AMI) was a severe type of cardiovascular disease and was defined as AMI diagnosed for the first time. Patients with first incident AMI among CAD patients have more cardiovascular risk factors (4). Risk factors management has been the corner-stone for prevention and therapy of first incident AMI.

Calcium, one of the most important cations, plays a pivotal role in biological processes such as cardiac contraction, blood pressure regulation, and blood coagulation (5,6). Maintaining calcium flux balance is particularly important for myocardium (7). Hypocalcaemia is a common electrolyte disturbance of critically ill patients, and it has been shown to predict poor outcomes (8,9). What is more, calcium intake decreased the incidence of myocardial infarction (10,11). On the other hand, high levels of serum calcium significantly increased the risk of CAD, in particular AMI (12). However, calcium intake within tolerable upper intake levels of 2000 to 2500 mg/d was not associated with cardiovascular disease in generally healthy adults (13).

The relationship between serum calcium and AMI continues to be controversial, which is confusing for the physician and has restricted its application in clinical practice. Although much work has been done to determine the relationship between them in Caucasian populations, few studies have been conducted in Chinese population. What is more, the effect of serum calcium on first incident AMI has not been addressed. In addition, previous studies did not consider the potential presence of multicollinearity among covariates, which did not entirely exclude confounding factors. In this study, a well-designed cross-sectional study was performed to assess the role of serum calcium in CAD patients with or without first incident AMI in our medical center.

## Methods

### The database and participants

This cross-sectional study was conducted from Jan 2014 to Dec 2016. All the participants were from our database, described in detail elsewhere (14).

The study was performed according to the Declaration of Helsinki. Patients were informed of the purpose and procedures of our study before giving their consent in written form.

To reduce the confounding factors in the study, patients with the following conditions were excluded: liver abnormality; kidney abnormality; malignant tumor; acute infection; parathyroid disease; thyroid abnormality; medical history including taking vitamin D; bisphosphonate, or diuretics within the past one month; the absence of serum calcium.

First incident AMI was defined as AMI diagnosed for the first time according to the third universal definition of myocardial infarction in 2012 without a history of AMI (15). CAD was diagnosed largely according to the coronary angiography (CAG) when the diameter stenosis was ≥50% with at least one main coronary artery or its major branch involved, or a history of revascularization by percutaneous coronary intervention or coronary artery bypass graft, or Angina pectoris. Angina pectoris was defined as effort substernal chest discomfort relieved by rest with dynamic changes in electrocardiogram.

### Laboratory analysis

Fasting (8h overnight) blood samples were collected on the second day morning after admission. Reagents for measuring calcium, sodium, potassium, phosphate, albumin, creatinine, low-density lipoprotein cholesterol (LDL-C), high-density lipoprotein cholesterol (HDL-C), apolipoprotein A (apo A), apolipoprotein (apo B), lipoprotein (a) (Lp (a)), total triglyceride (TG) and total cholesterol (TC) were obtained from Sekisui Diagnostic Ltd. An Olympus AU5400 analyzer was used to measure the biochemical markers. Calibrator and control reagents were performed according to the laboratory’s protocols.

### Statistical analysis

The Kolmogorov-Smirnov test was performed to analyze normality of continuous variables. As all the data failed to conform to a normal distribution, continuous variables were expressed as the median (interquartile range) (IQR). The Mann-Whitney U test was used to compare continuous variables. Categorical variables were shown as frequencies and percentages. To analyze these kinds of variables, the likelihood-ratio Chi-squared test was applied.

The categorization of serum calcium was performed based on the whole sample population. The serum calcium levels were divided into fifths and incorporated into regression models. The serum calcium levels of Quintile 1 (Q1)-Q5 were 2.34–2.95, 2.26–2.34, 2.18–2.26, 2.1–2.18, and 1.4–2.1 mmol/L. Q1 was used as a reference. The odds ratio (OR) for Q2, Q3, Q4, and Q5 were analyzed to compare to the reference.

Logistic regression models were performed among CAD patients with or without first incident AMI. Model 1: crude OR, no risk factors were adjusted. Model 2: partially adjusted OR for risk factors including age and sex. Model 3: fully adjusted OR for risk factors including age, sex, smoking, drinking, primary hypertension (PH), diabetes mellitus (DM), body mass index (BMI), ischemic stroke (IS-stroke), hemorrhagic stroke (HE-stroke), LDL-C, HDL-C, Lp (a), TG, serum albumin, and phosphate. Partially and fully adjusted models did not adjust sex in the subgroups divided by gender and did not adjust age in subgroups based on age. Multicollinearity was performed to quantify the potential confounding covariates. As a common rule, a variance inflation factor (VIF) >5 was considered for the presence of multicollinearity. Therefore, Apo A1/B ratio, Apo A, Apo B, and TC values were removed.

The interaction of serum calcium with other risk factors was examined by including 2 factor interaction terms between serum calcium on a continuous scale and other examined risk factors, one at a time, in the fully adjusted logistical regression model. I-squared was measured to quantify heterogeneity between stratifications.

All graphs were completed with STATA 14.0 and Illustrator CC. The final data were analyzed with STATA 14.0. *P*<0.05, which is two-sided, was considered to be statistically significant.

## Results

**Fig. 1:**
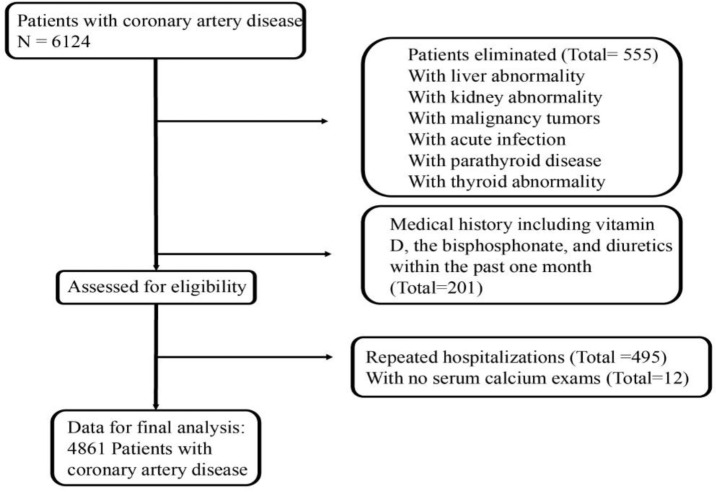
Flow chart of patient selection

There were 6,124 patients available for potential analysis, of which 555 were excluded due to the presence of the diseases described in the methods, 201 due to the medications described above, 495 due to repeat hospitalizations, and 12 due to the lack of serum calcium examination. Details are shown in the flow chart in Fig. 1.

### Characteristics of the population

This study included 1,609 patients with first incident AMI and 3,252 CAD patients without first incident AMI. A summary of the clinical characteristics of the population is shown in Table 1.

**Table 1:** Demographic characteristics

***Variables***	***All***	***With AMI***	***Without AMI***	***P-value***
N	(n=4861)	(n=1609)	(n=3252)	
Men (n, %)	3696(76.03)	1320(82.04)	2376(73.06)	<0.001
Age (yr)^a^	65(15)	62(19)	66(14)	<0.001
BMI (Kg/m^2^)^a^	24.5(3.8)	24.6(3.7)	24.5(3.8)	0.452
Vital signs				
SBP (mmHg)^a^	130(20)	124(29)	130(20)	<0.001
DBP (mmHg)^a^	78(15)	78(16)	78(13)	0.386
HR (bpm)^a^	72(16)	76(20)	70(15)	<0.001
Life sytles(n, %)				
Smoking				<0.001
Never	2242(46.12)	544(33.81)	1698(52.21)	
Past smoking	779(16.03)	178(11.06)	601(18.48)	
Current smoking	1840(37.85)	887(55.13)	953(29.31)	
Alcohol intake (n, %)				<0.001
Never	2863(58.90)	809(50.28)	2054(63.16)	
Past drinking	69(1.42)	14(0.87)	55(1.69)	
Current drinking	1929(39.68)	786(48.85)	1143(35.15)	
Past history (n, %)				
Is-stroke	327(6073)	83(5.16)	244(7.50)	0.002
He-stroke		14(0.87)	15(0.46)	0.09
PH	3211(66.06)	949(58.98)	2262(69.56)	<0.001
DM	1230(25.30)	385(23.93)	845(25.98)	0.121
Blood analysis				
TC (mmol/L)^a^	3.9(1.43)	4.29(1.35)	3.71(1.39)	<0.001
TG (mmol/L)^a^	1.31(0.95)	1.36(0.98)	1.29(0.93)	<0.001
Lp(a) (mg/L)^a^	120(227.15)	123(215.4)	118(233.9)	0.232
ApoA (g/L)^a^	1.24(0.29)	1.2(0.27)	1.26(0.31)	<0.001
ApoB (g/L)^a^	0.82(0.38)	0.92(0.35)	0.76(0.36)	<0.001
ApoA1/B^a^	1.5(0.8)	1.3(0.6)	1.6(0.8)	<0.001
HDL-C (mmol/L)^a^	1.07(0.34)	1.07(0.33)	1.07(0.34)	0.943
LDL-C (mmol/L)^a^	2.31(1.22)	2.67(1.15)	2.1(1.16)	<0.001
Serum albumin (g/L)^a^	40.6(5)	40(5.3)	41(4.7)	<0.001
Creatinine (μmol/L)^a^	72(22)	72(21.2)	72.3(22)	0.415
Serum sodium (mmol/l)^a^	142(1.4)	141.3(3.7)	142.7(3)	<0.001
Serum potassium (mmol/l)^a^	3.9(0.43)	3.9(0.4)	3.9(0.46)	0.569
Serum phosphate (mmol/l)^a^	1.05(0.27)	1.15(0.28)	1.1(0.25)	<0.001
Serum calcium (mmol/L)^a^	2.22(0.2)	2.18(0.21)	2.24(0.19)	<0.001
Medications (n, %)				
Aspirin	4116(84.67)	1449(90.06)	2667(82.01)	<0.001
Clopidogrel	3346(68.83)	1448(90)	1898(58.36)	<0.001
ACEI/ARB	3683(75.77)	1078(67)	2605(80.1)	<0.001
Beta-blockers	3515(72.31)	1239(77)	2276(70)	<0.001
CCB	922(18.97)	109(6.77)	813(25)	<0.001
Nitrates	1521(31.29)	269(16.72)	1252(38.5)	<0.001
Statins	4331(89.1)	1453(90.3)	2878(88.5)	0.055

AMI: acute myocardial infarction, BMI: body mass index, SBP: systolic blood pressure, DBP: diastolic blood pressure, HR: heart rate, IS-stroke: ischemic stroke, HE-stroke: hemorrhagic stroke, PH: primary hypertension, DM: diabetes mellitus, TC: total cholesterol, TG: Triglycerides, Lp (a): Lipoprotein (a), Apo A: Apolipoprotein A, Apo B: Apolipoprotein B, LDL-C: Low-density lipoprotein cholesterol, HDL-C: High-density lipoprotein cholesterol, ACEI/ARB: Angiotensin converting enzyme inhibitor/angiotensin 2 receptor inhibitor, CCB: Calcium channel blocker. Values are medians (interquartile range) or numbers (percentage).

Categorical variables were compared with χ^2^ test.

a Data with skewed distribution are shown as median (IQR). Mann-Whitney U test was performed

The female patients were less prone to AMI (17.96%). The average age of the whole sample population was 65 yr old. The two groups presented a similar BMI. CAD patients with first incident AMI showed a higher percentage of current smoking (55.13% vs 29.31%, *P*<0.001) and current drinking (48.85% vs 35.15%, *P*<0.001) compared with those without first incident AMI. CAD patients without first incident AMI showed a greater prevalence for both IS-stroke and primary hypertension. Participants with AMI exhibited higher levels of TC, TG, Apo B, LDL-C, and phosphate but lower levels of Apo A, Apo A1/B, serum albumin, and sodium. A significant statistical difference in serum calcium (2.18 (0.21) vs 2.24 (0.19) mmol/L, *P*<0.001) was observed. Patients with first incident AMI were more likely to be treated with aspirin, clopidogrel, beta-blockers, and statins.

### Association between serum calcium levels and first incident AMI on a continuous scale

The mean of serum calcium was 2.22 mmol/L, and the IQR was 0.2 mmol/L. On a continuous scale, multiple logistic regression models showed that a 1 standard deviation (SD) (−0.15 mmol/L) decrease of serum calcium levels was associated with a crude adjusted OR of 1.45, 95% CI (1.36–1.54), a partially adjusted OR of 1.50, 95% CI (1.41–1.60) and a fully adjusted OR of 1.32, 95% CI (1.22–1.42) for first incident AMI in all patients. Slightly enhanced results were showed in women. Similar, but decreased results were displayed in men (Table 2).

**Table 2:** OR of per 0.15 mmol/L lower serum calcium levels for first incident AMI

	***Men***		***Women***		***Total***	

	OR (95%CI)	P values	OR (95%CI)	P values	OR (95%CI)	*P* values
Model 1	1.40 (1.31–1.50)	*P*<0.0001	1.58 (1.37–1.83)	*P*<0.0001	1.45 (1.36–1.54)	*P*<0.0001
Model 2	1.50 (1.39–1.61)	*P*<0.0001	1.57 (1.36–1.81)	*P*<0.0001	1.50 (1.41–1.60)	*P*<0.0001
Model 3	1.30 (1.20–1.42)	*P*<0.0001	1.38 (1.17–1.62)	*P*<0.0001	1.32 (1.22–1.42)	*P*<0.0001

Model 1: Crude

Model 2: adjusted for age and sex

Model 3: adjusted for age, sex, drinking, smoking, primary PH, DM, BMI, IS-stroke, HE-stroke, LDL-C, HDL-C, Lp(a), TG, serum albumin, and phosphate

### Association between serum calcium levels and first incident AMI on a categorical scale, grouped by sex

**Fig. 2:**
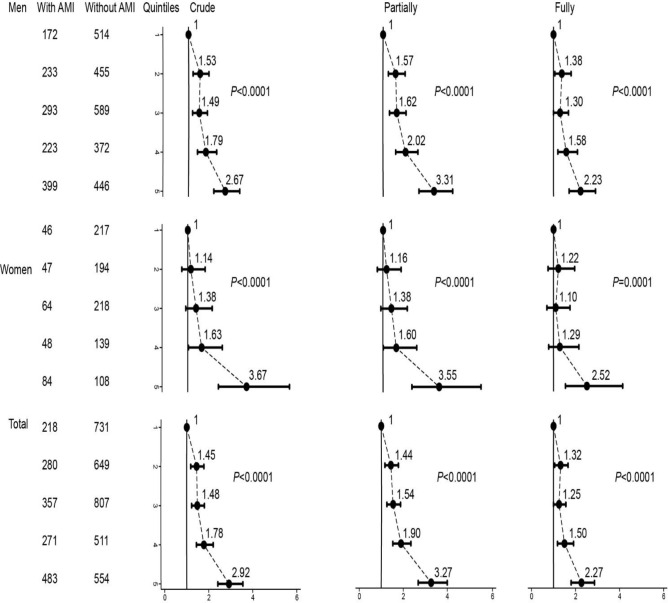
On a category scale, crude, partially and fully adjusted OR for first incident AMI among CAD patients, grouped by sex. Q1 (as reference), Q2, Q3, Q4, and Q5 represent increased quintile of serum calcium concentrations. Black dots represent point estimates, and error bars, 95% confidence intervals. *P* values are for trend test

Serum calcium concentrations were divided into fifths (Q1-Q5: 2.34–2.95, 2.26–2.34, 2.18–2.26, 2.1–2.18, and 1.4–2.1 mmol/L). Crude, partially and fully adjusted logistic regression models showed that the percentage of first incident AMI increased, as serum calcium concentrations increased across its Quintiles. With increasing quintiles, fully adjusted OR for first incident AMI were 1.38, 95% CI (1.06–1.80) for Q2 (Q1 as reference), 1.3 (1.00–1.68) for Q3, 1.58 (1.20–2.09) for Q4, and 2.23 (1.71–2.91) for Q5 in men; the equivalents in women were 1.22 (0.75–1.96), 1.10 (0.7–1.75), 1.29 (0.78–2.15), and 2.52 (1.54–4.14); and 1.32 (1.05–1.66), 1.25 (1.00–1.56), 1.50 (1.18–1.91), and 2.27 (1.8–2.86) in total. Interestingly, OR showed statistically significant difference in Q2-Q5 in crude, partially, and fully adjusted models. Even after adjusting for the potential confounding covariates, the tendency persisted with an OR of 2.27 in Q5 (Fig. 2).

### Association between serum calcium levels and first incident AMI on a categorical scale, grouped by age

**Fig. 3:**
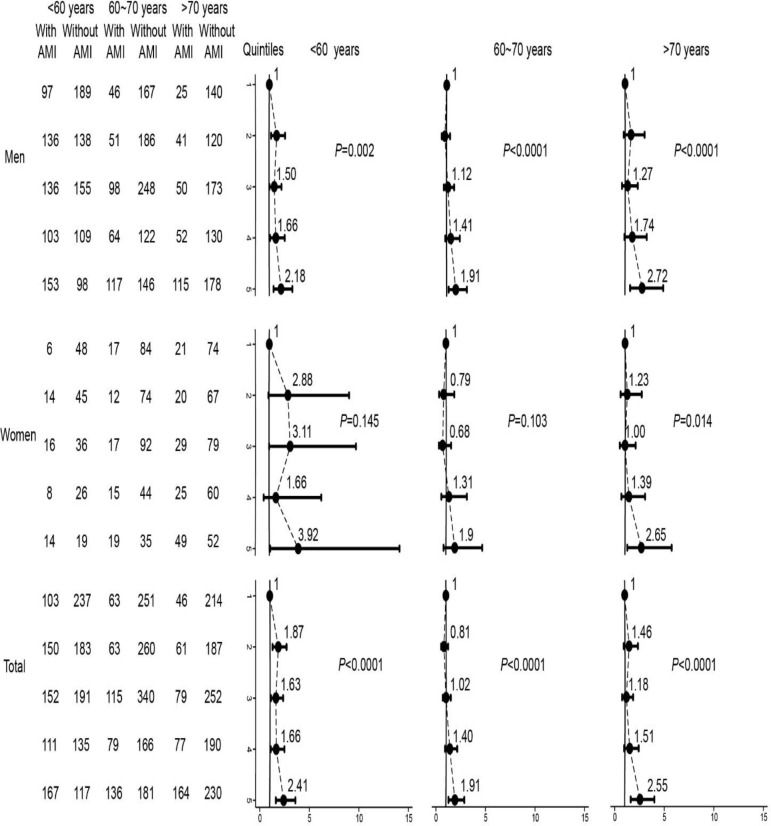
On a category scale, crude, partially and fully adjusted OR for first incident AMI among CAD patients, grouped by age. Q1 (as reference), Q2, Q3, Q4, and Q5 represent increased quintile of serum calcium concentrations. Black dots represent point estimates, and error bars, 95% confidence intervals. *P* values are for trend test

Fully adjusted logistic regression models showed that lower calcium concentration increased first incident AMI at different ages in total. With increasing quintiles, fully adjusted OR for first incident AMI were 1.87 (95% CI, 1.29–2.7) for Q2 (reference to Q1), 1.63 (1.13–2.36) for Q3, 1.66 (1.11–2.49) for Q4, and 2.41 (1.62–3.59) for Q5 in patients younger than 60 yr old; the equivalents were 0.81 (0.53–1.23), 1.02 (0.70–1.50), 1.40 (0.92–2.15), and 1.91 (1.27–2.86) in patients between 60–70 yr old, and 1.46 (0.91–2.34), 1.18 (0.75–1.86), 1.51 (0.95–2.42), and 2.55 (1.62–4.00) in patients older than 70 yr old (Fig. 3).

### Risks of first incident AMI per 0.15 decrease of serum calcium levels

**Fig. 4:**
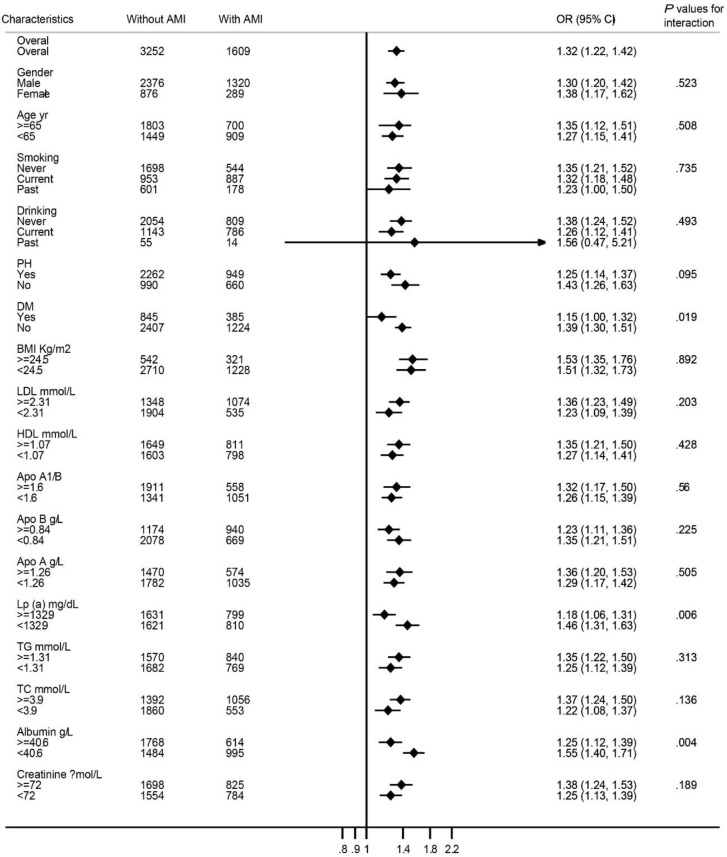
On a continuous scale, fully adjusted OR of per 1SD (0.15 mmol/L) lower serum calcium concentrations for first incident AMI among CAD patients, grouped by characteristic stratification, dichotomously, trichotomous or medially. Black dots represent point estimates, and error bars, 95% confidence intervals. *P* values are for the interaction

Risk factors were stratified by characteristics, dichotomously, trichotomous or medially and included into the fully adjusted model. A significant interaction was found between hypocalcemia and DM, serum albumin, and Lp (a) on the risk of first incident AMI (Fig. 4).

## Discussion

This is a cross-sectional study including 4861 patients showing that low serum calcium levels were associated with increased first incident AMI. The main finding of this study is that serum calcium concentrations were significantly decreased in patients with newly diagnosed AMI. The OR of having first incident AMI gradually increased following the increasing quintiles of serum calcium. Further, the results were only marginally attenuated after fully adjustment for potential confounding factors. DM, Lp(a), and serum albumin could modify the risk of serum calcium concentrations for first incident AMI. Collectively, serum calcium might be a risk factor associated with the development of first incident AMI.

Information regarding the role of serum calcium levels in AMI remains controversial. The association between serum calcium and first incident AMI in patients grouped by age and sex has not been reported. What is more, there is no related study showing the relationship among CAD patients in Chinese Han population. In our study, compared to patients without first incident AMI, serum calcium was significantly decreased in patients with first incident AMI. Figure 2 showed that the OR for the female participants varies largely, especially at low serum calcium concentrations, as the female participants were less prone to first incident AMI. The OR for Q2-Q5 had statistic difference in men and total. Fully adjusted logistic regression results showed that participants in the Q5 of serum calcium increased approximately 2.3-fold possibility to suffer from first incident AMI. AMI patients had lower serum calcium levels than patients without AMI in a single medical center (4). Multiple logistic regression analysis revealed that serum calcium was one of the independent factors that correlated with AMI. Our results were consistent with previous findings. However, the study did not perform multiple logistic regression analysis in different concentrations of serum calcium in subgroups. Our results were also similar to parts of results from the Tromso study, although serum calcium levels were higher in men with a history of AMI. The Tromso study revealed the relationship between serum calcium and history of AMI but not AMI, which may account for the different results (16). The relationship between low serum calcium levels and first incident AMI was further detected in patients grouped by sex and age after full adjustment. It is not surprisingly that OR for female patients in different age categories varied obviously and did not reach statistically significant difference except for patients older than 70 yr, because female patients were ever less. Despite the OR attenuated significantly when patients grouped by age, however, lower serum calcium would increase the first incident AMI. Few studies have focused on the interaction effects. Our results also demonstrated that DM, Lp (a), and serum albumin may modify the risk of serum calcium levels for AMI. Other studies also confirmed these results (17–19).

To the best of our knowledge, there are three primary forms of calcium in serum. Approximately 50% of serum calcium exists in the free or ionized form, which regulates cell function and systems physiology, about 40% binds to albumin, which reflects the serum albumin levels, and the remaining 10% is bound to anions such as phosphate, bicarbonate, and lactate (20). Although low serum calcium concentrations might increase first incident AMI, the mechanisms remain unclear. Possible mechanisms were described in detail. Serum calcium tightly tied to cardiovascular risk factors. First of all, the serum calcium concentrations of the hypertensive group were lower than that of the normotensive group in older men in East China (21). Serum calcium participates in vascular smooth muscle cell contractility and inhibits renin secretion, which mediates blood pressure. High serum calcium has a protective function in hypertension. Secondly, Serum calcium interfered with lipid absorption through binding to bile acids and fatty acid. Calcium supplementation was increased HDL cholesterol levels and decreased LDL cholesterol levels at meanwhile (22). Lastly, dietary calcium from dairy foods could decrease the accumulation of body fat, which decreased the prevalence of DM (18). The presence of classical independent risk factors, such as Lp (a) (14), hypertension, DM, smoking, and hyperlipidemia, increases the incidence of AMI (4).

Despite relatively comprehensive clinical data collection and consecutive patient recruitment, our study still has some limitations. Firstly, this study did not measure ionized calcium, which is the physiological fraction of calcium. Some studies have used serum albumin to correct serum calcium, but there is no clear consensus (23–25). As serum albumin was also included in the fully adjusted logistic regression model in this study, the interference of the serum albumin on serum calcium could be eliminated. Secondly, calcium metabolism is extremely complex and is affected by many factors. Although diuretics were excluded from further analysis and adjustments of other markers were selected carefully, it remains possible that other factors may involve in the increased incidence of first incident AMI. Last but not least, this is a single-center cross-sectional study and recruited exclusively from a Chinese Han population. To validate these results, clinical trials should be further performed in other ethnic groups.

## Conclusion

Lower serum calcium levels are associated with a greater risk of first incident AMI both in age and sex categories. It still remains unclear how or when to intervene in conditions of low serum calcium to minimize first incident AMI risk among CAD patients.

## Ethical considerations

Ethical issues (Including plagiarism, informed consent, misconduct, data fabrication and/or falsification, double publication and/or submission, redundancy, etc.) have been completely observed by the authors.
